# Psychometric Validation of the Greek Versions of the Generic Scale of Phubbing (GSP) and the Generic Scale of Being Phubbed (GSBP) Among Nursing Personnel

**DOI:** 10.1155/nrp/5200164

**Published:** 2026-08-27

**Authors:** Georgios Manomenidis, Elena Vasileiou, Vasiliki Georgousopoulou

**Affiliations:** ^1^ Department of Nursing, Democritus University of Thrace, Alexandroupoli, Greece, duth.gr; ^2^ Department of Occupational Health Services, University Hospital of Alexandroupoli, Alexandroupoli, Greece

**Keywords:** being phubbed, nursing population, phubbing, psychometric properties

## Abstract

**Background:**

As everyday attention is routinely pulled towards smartphones with almost everyone engaging in it to some extent the investigation of phubbing (snubbing someone by focusing on a phone) and being phubbed (experiencing that snub) has become increasingly important for understanding how technology shapes face‐to‐face interaction.

**Aim:**

To evaluate the psychometric properties of the Greek Generic Scale of Phubbing (GSP) and the Generic Scale of Being Phubbed (GSBP) in a nursing population.

**Methods:**

A cross‐sectional psychometric validation study conducted with 406 nursing personnel recruited from one tertiary and five secondary hospitals in Greece (July to September 2025). Participants completed the 15‐items GSP, the 22 items GSBP, and the Smartphone Addiction Scale–Short Version (SAS‐SV). Exploratory and confirmatory factor analyses were used while internal consistency was assessed with McDonald’s *ω*, and convergent validity with Spearman correlations with SAS‐SV.

**Results:**

EFA supported a four‐factor solution for the GSP and a three‐factor solution for the GSBP. CFA fit was adequate for the GSP and marginal‐to‐acceptable for the GSBP. Reliability was good to excellent, while the CFA‐derived AVE/CR indices supported convergent validity, and Fornell–Larcker comparisons supported discriminant validity. SAS‐SV correlated moderately with GSP total and more weakly with GSBP total.

**Conclusion:**

The Greek GSP and GSBP yield reliable and valid scores for assessing phubbing and being phubbed among nursing personnel, supporting future research on technology‐related disruption in clinical communication and teamwork.

## 1. Introduction

In an era of constant connectivity, subtle yet regular distractions from the smartphone increasingly intrude upon face‐to‐face interactions, quietly affecting the flow of conversation, focus, and relationship quality [1]. Although each interruption may last only a few seconds, their cumulative effect can build up a broader feeling of disconnection and exclusion. Smartphones have become integral tools for fulfilling communication needs, accessing everyday information, and engaging with social media platforms, thereby fostering constant connectivity and interaction [2]. These functions, combined with smart push notifications and personalized content delivery, can create a compulsive urge to check one’s devices, diverting attention from face‐to‐face interactions, leading to a dependent relationship between individuals and their smartphone [3, 4].

The COVID‐19 pandemic fostered a uniquely conducive atmosphere for the intensification of these dynamics, especially in the health sector. Increased uncertainty, job instability, and health‐related stress were observed in frontline healthcare personnel experiencing significant distress, burnout, and apprehension [5, 6]. The fear of infection, extensive working hours, and swiftly altering protocols resulted in an environment of ongoing psychological stress [7, 8]. Evidence from ICU nurses during the pandemic has also shown high stress and low resilience, highlighting the psychological strain experienced by frontline nursing personnel [9]. In these situations, digital devices frequently served as coping mechanisms, providing connection, distraction, or comfort while also leading to “technoference”—technology‐based interruptions during important interactions [10].

Within this broader context of “technoference”, an increasing number of studies have highlighted several psychosocial factors that contribute to phubbing—the act of snubbing someone in a social setting by looking at one’s phone instead of paying attention [11]. Challenges in controlling smartphone use, increased fear of missing out, and trait anxiety are reliable indicators of obsessive checking, rapid responses, and excessive dependence on digital rewards [12]. The corollary experience, being phubbed, describes being on the receiving end of this social disregard [13]. When translated into the clinical nursing environment, these behaviors create a dichotomy of effects that impact both the perpetrator and the recipient of the phubbing act. For the nurse who engages in phubbing, the primary consequences manifest as a degradation of professional practice, including significant attentional deficits that elevate patient safety risks and a depersonalization of care that corrodes the therapeutic relationship [14]. The act of prioritizing a device over a present colleague or patient inherently compromises the nurse’s immediate professional responsibilities. Conversely, for the nurse who is being phubbed, the effects are centered on professional morale and interpersonal workplace dynamics [11]. When a nurse is phubbed by a colleague, and especially by a supervisor, it fosters a professional environment characterized by perceived disrespect and psychological devaluation. This experience has been empirically linked to a marked decline in work engagement, diminished job performance, and an erosion of intrinsic motivation, as it undermines the foundations of collegial trust and psychological safety [15]. Therefore, the act of phubbing in a clinical setting does not merely represent a momentary distraction but rather a significant professional slight that degrades team cohesion and the overall standard of care.

Emerging evidence indicates that phubbing is a common pattern across social, educational, and healthcare‐related contexts. Studies have reported frequent phubbing among adolescents and university students, while healthcare‐focused evidence shows that smartphone use during clinical practice is also relevant in nursing settings [16, 17]. Among nursing students in South Korea, smartphone use and distraction during clinical practice were reported, and among hospital registered nurses in the United States, personal mobile phone use during work was common and was perceived by many nurses as potentially affecting patient care [18–20]. These findings support the relevance of examining phubbing‐related behaviors in nursing personnel.

Several instruments have been developed to assess phubbing and being phubbed across different relational and social contexts. Some measures focus on specific relationships, such as romantic partners, parents, supervisors, peers, or workplace interactions, whereas others provide broader assessments of perceived phubbing [21–25]. Among these instruments, the Generic Scale of Phubbing (GSP) and the Generic Scale of Being Phubbed (GSBP) were developed to assess both the enactment of phubbing and the experience of being phubbed across a wide range of everyday interactions, making them suitable for use beyond a single relational context [26].

The GSP and GSBP have also been used in clinical and health‐related samples, including nurses and nursing students, to quantify both phubbing behavior and the experience of being phubbed [12, 27–29]. The GSP/GSBP pair was selected for the present study because it captures complementary perspectives on the same interactional phenomenon: phubbing as a behavior and being phubbed as the recipient’s experience. In addition, the GSP assesses theoretically relevant dimensions such as nomophobia, interpersonal conflict, self‐isolation, and problem acknowledgement, while the GSBP captures perceived norms, feeling ignored, and interpersonal conflict [26]. Nomophobia, commonly understood as discomfort, nervousness, or anxiety associated with being out of contact with one’s mobile phone, has been identified as one of the psychological dimensions underlying phubbing behavior [30]. Although the GSP has recently been adapted and psychometrically evaluated in Greek nonclinical samples [31], to the best of our knowledge, the GSBP has not been validated in Greek, and no study has examined both instruments among Greek nursing personnel. This gap limits the availability of robust tools for assessing phubbing and being phubbed among Greek healthcare professionals and provides rationale for the present psychometric validation of the Greek versions of the GSP and the GSBP in a nursing population. The present study aimed to translate, culturally adapt and evaluate the psychometric properties of the GSP and the GSBP among nurses in Greece.

## 2. Methods

### 2.1. Study Design

This study was designed as a cross‐sectional psychometric validation study aimed at translating, culturally adapting, and evaluating the psychometric properties of the Greek versions of the GSP and the GSBP in a nursing population.

### 2.2. Sample and Data Collection

A convenience sample of 406 nursing personnel working in one tertiary and five secondary hospitals in Northern Greece was recruited between July and September 2025 to complete a self‐completed paper‐based questionnaire. Recruitment was facilitated by members of the research team who disseminated the description of the study via multiple channels, including printed posters displayed in nursing stations of hospitals and staff portals. Interested personnel received further information and were provided with an anonymous questionnaire enclosed with an envelope that was asked to return sealed in a general box placed in every department. Eligibility criteria included a permanent status of employment and at least 1 year of working experience. Completion time was approximately 15–20 min.

### 2.3. Sample Size

The sample size was planned to ensure stable factor solutions and precise validity estimates. For the GSP (15 items) and GSBP (22 items) estimated with DWLS/WLSMV on polychoric correlations, guidelines recommend *N* ≥ 300 [32]; the study’s final *N* = 406 exceeding this. For convergent validity, *N* = 406, provides > 99% power for *ρ* = 0.30, ≈ 98% for *ρ* = 0.20, and ≈ 86% for *ρ* = 0.15 (*α* = 0.05, two‐tailed). Overall, the study was well powered for the primary psychometric aims (factor structure, reliability, and convergent validity).

### 2.4. Measures

#### 2.4.1. Demographic and Occupational Characteristics

A demographic and occupational characteristics questionnaire was constructed for the study that included age, gender, professional category (nurse or nurse assistant), family status, years of professional experience, and department/unit of employment.

#### 2.4.2. The GSP

The GSP was used to assess phubbing behavior [26]. The scale consists of 15 items rated on a 7‐point Likert scale with verbal anchors at every point (1 = Never and 7 = Always). It comprises of four subscales: nomophobia (fear of being without the phone; 4 items), interpersonal conflict (phone use causing conflict with others; 4 items), self‐isolation (using the phone to withdraw from social interaction; 4 items), and problem acknowledgement (recognition of problematic phone use; 3 items). The GSP measure is scored by summing all item scores with higher scores corresponding to more frequent phubbing.

#### 2.4.3. The GSBP

The GSBP is a psychometric instrument that assesses individuals’ experience of being ignored or dismissed by others during social interactions due to the others’ engagement with their smartphones [26]. The scale consists of 22 items, answered using a 7‐point Likert scale where 1 corresponds to “Never” and 7 to “Always”. It comprises of three subscales: perceived norms, feeling ignored, and interpersonal conflict. Scores are interpreted according to the sum of responses to each item, with higher scores corresponding to greater perception of being ignored.

#### 2.4.4. The Smartphone Addiction Scale–Short Version (SAS‐SV).

The SAS‐SV [33] is a psychometric tool that assesses problematic or addictive smartphone use, particularly among adolescents and young adults. It is a brief version of the original SAS and consists of 10 items rated on a 6‐point Likert scale where 1 corresponds to “Strongly disagree” to 6 that corresponds to “Strongly agree”, yielding a total score ranging from 10 to 60, with higher scores indicating a greater risk of smartphone addiction. The scale evaluates key aspects such as loss of control over use, sleep disturbances, functional impairment, and emotional dependence on the device. In the present study, the Greek version of the SAS‐SV was used to assess convergent validity. In the current sample, the SAS‐SV demonstrated good internal consistency with McDonald’s *ω* = 0.828. The Greek SAS‐SV has recently shown acceptable psychometric properties among nursing students in Greece [34] and was therefore considered appropriate for research use in Greek‐speaking populations.

#### 2.4.5. Translation and Cultural Adaptation

All three instruments (GSP, GSBP, and SAS‐SV) were translated and culturally adapted following the guidelines proposed by Beaton [35] for cross‐cultural adaptation of self‐report instruments. Two independent bilingual translators outside the research team, performed the forward translations from English into Greek. The two Greek versions were compared and reconciled into a single preliminary version. A blind back‐translation was then performed by two bilingual translators who had not seen the original instruments. An expert committee consisting of 10 members reviewed all versions. The committee included nursing academics, and clinical nurses. Discrepancies were resolved through discussion until consensus was reached, with emphasis on semantic, idiomatic, experiential, and conceptual equivalence. The prefinal Greek versions were pilot tested through cognitive debriefing interviews with 10 nursing personnel, including six nurses and four nurse assistants; seven participants were female and three were male. Participants were asked to comment on item clarity, wording, cultural relevance, and response options. Minor wording refinements were incorporated before finalizing the instruments. These 10 participants were excluded from the final analytic sample. The interviews lasted approximately 5–10 min each. The final Greek versions of the GSP, GSBP, and SAS‐SV are available from the corresponding author upon reasonable request.

### 2.5. Statistical Analysis

Item distributions were checked (means, standard deviations, skewness, and kurtosis). Given deviations from normality at the item level, items were treated as ordinal throughout. Exploratory factor analysis (EFA) used principal axis factoring with oblique rotation (Oblimin); factorability was evaluated via KMO, Bartlett’s test, the determinant, interitem correlations, and communalities. Factor retention was guided by eigenvalues and the scree plot. Confirmatory factor analysis (CFA) was conducted on polychoric correlations with a DWLS/WLSMV estimator; we reported *χ* ^2^/df, CFI, TLI, RMSEA, SRMR, and standardized loadings. Model fit was evaluated using commonly reported fit indices. CFI and TLI values close to or above 90 were considered acceptable and values close to or above 95 are good; RMSEA and SRMR values below 08 were considered acceptable, while values close to or below 06 indicated a better fit. Convergent validity was examined via Spearman’s *ρ* between GSP/GSBP totals (and subscales) and SAS‐SV totals with 95% bootstrap CIs. McDonald’s *ω* was utilized as evidence of the reliability of measurements obtained from measurement tools. Convergent reliability and discriminant validity were further assessed using AVE and composite reliability (CR) from CFA standardized loadings; Fornell–Larcker was evaluated by comparing √AVE to interfactor correlations. Data management, descriptive statistics, and EFAs were performed in IBM SPSS Statistics (Version 27). CFAs were conducted in JASP (Version 0.17) using the SEM/CFA module (lavaan engine).

## 3. Results

A total of 406 nursing personnel participated in the study. The mean age of the sample was 46.6 years (± 8.31). Most participants were female (88.5%), who lived with their family/partner (84.4%), were nurses (69.1%), and were working in general medical wards (54%). The demographic and occupational characteristics of the participants are displayed in Table 1.

**TABLE 1:** Demographic and professional characteristics.

Variables	*n* (%)
*Gender*	
Male	45 (11.5%)
Female	361 (88.5%)
Age^a^	46.6 (8.31)

*Living arrangement*	
Live alone	76 (15.6%)
Live with my partner/family	330 (84.4%)

*Professional category*	
Nurses	270 (69.1%)
Nurse assistants	136 (30.9%)
Working experience^a^	19 (10.4)

*Working department*	
General medical wards	219 (54%)
Critical/Interventional Units	187 (46%)

^a^ mean (SD).

### 3.1. The GSP

The descriptive statistics of the GSP are presented in Table 2. The observed items’ scores range from 1.56 (± 0.85) and 4.03 (± 1.76); the skewness values range from −0.076 to 2.102 and the kurtosis values from −0.066 to 5.549. The distribution of responses for the 15 items did not meet the assumption of normality (positive skew and kurtosis across many items).

**TABLE 2:** Descriptive statistics for the GSP.

Items	Mean	Std	Skewness	Kurtosis
GSP1	3.56	1.57	0.269	−0.524
GSP2	3.16	1.61	0.336	−0.779
GSP3	4.03	1.76	−0.076	−1.001
GSP4	3.24	1.64	0.549	−0.573
GSP5	1.78	1.03	1.629	2.974
GSP6	1.85	1.19	1.819	3.584
GSP7	1.60	0.90	1.757	3.392
GSP8	1.79	1.15	2.102	5.549
GSP9	1.78	1.04	1.151	0.224
GSP10	1.56	0.85	1.680	2.514
GSP11	1.62	0.95	1.704	2.461
GSP12	1.83	1.06	1.715	0.625
GSP13	2.73	1.44	0.617	−0.066
GSP14	2.30	1.39	1.240	1.410
GSP15	2.57	1.43	0.671	−0.301

Preliminary analyses evaluated the factorability of the 15 GSP items to determine the appropriateness for EFA. The Kaiser–Meyer–Olkin (KMO) measure of sampling adequacy was 0.842 (*p* < 0.001), indicating excellent sampling adequacy. Bartlett’s test of sphericity was statistically significant (*χ* ^2^ = 2,827.647, *p* < 0.001). The determinant of the item correlation matrix was 0.001, indicating no problematic multicollinearity, while communalities exceeded 0.30 (range 0.457–0.762). Based on the eigenvalue pattern (*λ* = 5.45, 2.30, 1.29, 1.20) and scree, 4 factors were retained and extracted for principal axis factoring. After extraction, the first four factors accounted for 57.6% of the common variance (33.684%, 12.682%, 6.260%, and 5.023%). The oblique rotation (8 iterations) produced a clear simple structure: Factor 1 (Nomophobia) loaded on GSP10, GSP11, GSP9, GSP12 (*λ* = 0.668–0.830); Factor 2 (Interpersonal conflict) on GSP1, GSP2, GSP4, GSP3 (*λ* = 0.600–0.873); Factor 3 (self‐isolation) on GSP5, GSP6, GSP7, GSP8 (*λ* = 0.462–0.852); and Factor 4 (problem acknowledgement) on GSP15, GSP13, GSP14 (*λ* = 0.603, 0.691). Inter factor‐correlations were modest (≈ 0.24–0.47), consistent with oblique solution. On this basis, no items required removal, and the fourth‐factor solution was retained for reliability and subsequent confirmatory analyses. The factor loadings are presented in Table 3.

**TABLE 3:** Exploratory factor analysis: Rotated factor loadings for the generic scale of phubbing (GSP).

Items	Nomophobia	Interpersonal conflict	Self‐isolation	Problem acknowledgement
GSP10	0.817			
GSP11	0.814			
GSP9	0.668			
GSP12	0.622			
GSP1		0.874		
GSP2		0.768		
GSP4		0.714		
GSP3		0.591		
GSP5			0.852	
GSP6			0.823	
GSP8			0.570	
GSP7			0.462	
GSP15				0.691
GSP13				0.629
GSP14				0.603

A four‐factor model for the 15 GSP items showed adequate overall fit: *χ* ^2^ (84) = 280.54, *p* < 0.001; CFI = 0.94, TLI = 0.898, RMSEA = 0.082, SRMR = 0.0503. All standardized loadings were positive and significant (*p* < 0.001): Factor 1 = 0.668–0.827. (GSP10, GSP11, GSP9, and GSP12); Factor 2 = 0.582–0.875 (GSP1, GSP2, GSP4, and GSP3); Factor 3 = 0.591–0.899 (GSP5, GSP6, GSP7, and GSP8); Factor 4 = 0.679–0.766 (GSP15, GSP13, and GSP14). Standardized residual variances ranged roughly .19–66 consistent with the loading magnitudes. Modification indices indicated a small number of potential cross‐loadings (i.e., GSP12 on Factor 4; GSP6/GSP8 on Factor 1; GSP13 on Factors 2/3) and some within‐factor residual covariances; however, because these changes lacked sufficiently strong theoretical justification, we retained the prespecified simple‐structure model. The internal consistency of the scale was good (total *ω* = 0.854). McDonald’s *ω* subscale reliability was also strong, with values of 0.835 for “nomophobia”, 0.829 for “interpersonal conflict”, 0.831 for “self‐isolation” and acceptable 0.770 for “problem acknowledgement”. Regarding convergent validity, smartphone addiction measured with SAS‐SV and correlated positively with overall phubbing (GSP), *ρ* = 0.595, 95% CI [0.527,0.656], *p* < 0.001. SAS‐SV also showed moderate positive associations with the four GSP factors: nomophobia *ρ* = 0.463 95% CI [0.381,0.539], interpersonal conflict *ρ* = 0.461 95% CI [0.378,0.536], self‐isolation *ρ* = 0.473 95% CI [0.391,0.544], and problem acknowledgement *ρ* = 0.528 95% CI [0.451,0.596], all *p* < 0.001 (two‐tailed). The pattern and magnitudes (≈ 0.46–0.60) indicate moderate convergence between phubbing and smartphone addiction constructs. Using the standardized loadings from the CFA, the four GSP factors showed strong convergent evidence: AVE = 0.59–0.69 and CR = 0.81–0.90. Discriminant validity was supported by the Fornell–Larcker criterion: the diagonals √AVE = 0.770–0.833 exceeded all interfactor correlations (largest *r* = 0.694) indicating that each factor shares more variance with its own indicators than with other constructs (Table 4).

**TABLE 4:** GSP descriptive statistics, average variance extracted, and reliability analysis.

Instrument/subscale	*N*	M ± SD	AVE	√AVE	CR	*ω*
GSP total	406	35.44 ± 11.50	—	—	—	0.854
Nomophobia	406	6.80 ± 3.21	0.694	0.833	0.900	0.835
Interpersonal conflict	406	14.00 ± 5.37	0.607	0.779	0.858	0.829
Self‐isolation	406	7.03 ± 3.47	0.649	0.806	0.880	0.831
Problem acknowledgement	406	7.61 ± 3.55	0.593	0.770	0.814	0.770

*Note: N*, frequencies; M, mean; *ω*, McDonald’ *ω*.

Abbreviations: AVE, average variance extracted; √AVE, average variance square roots; CR, composite reliability; SD, standard deviation.

### 3.2. The GSBP

Descriptive statistics for the GSBP are shown in Table 5. Item means ranged from 2.19 (± 1.40) and 4.69 (± 1.11); skewness ranged from −1.123 to 1.030 and kurtosis from −0.938 to 1.552. Accordingly, the distribution of responses for the 22 items deviated from normality.

**TABLE 5:** Descriptive statistics for the GSBP.

Items	Mean	SD	Skewness	Kurtosis
GSBP1	4.37	1.14	−0.866	0.541
GSBP2	4.69	1.11	−1.223	1.552
GSBP3	4.56	1.22	−0.955	0.297
GSBP4	4.01	1.27	−0.319	−0.610
GSBP5	3.55	1.35	−0.097	−0.824
GSBP6	3.63	1.31	−0.341	−0.717
GSBP7	3.72	1.38	−0.188	−0.901
GSBP8	3.54	1.44	−0.136	−0.938
GSBP9	3.70	1.27	−0.103	−0.624
GSBP10	3.04	1.25	0.084	−0.730
GSBP11	2.88	1.26	0.342	−0.605
GSBP12	2.92	1.29	0.249	−0.744
GSBP13	2.79	1.31	0.310	−0.834
GSBP14	2.71	1.23	0.452	−0.520
GSBP15	2.80	1.33	0.456	−0.574
GSBP16	2.58	1.27	0.710	−0.117
GSBP17	2.58	1.23	0.503	−0.357
GSBP18	2.67	1.48	0.516	−0.737
GSBP19	2.19	1.40	1.030	0.041
GSBP20	2.66	1.43	0.460	−0.768
GSBP21	2.44	1.35	0.688	−0.451
GSBP22	2.44	1.37	0.501	−0.906

Abbreviation: SD, standard deviation.

Preliminary testing supported the factorability of the 22 GSBP items. Sampling adequacy was excellent (KMO = 0.930), and Bartlett’s test of sphericity was statistically significant (*χ* ^2^ = 8,281.346, *p* < 0.001). The correlation matrix determinant was 7.78 × 10^−10^, reflecting very high interitem correlations; together with the high KMO and significant Bartlett’s test, this justified proceeding to extraction. Communalities after extraction were all ≥ 0.459 (range 0.459–0.838). Based on the eigenvalue pattern (*λ* = 10.93, 3.32, 1.54) and the scree plot, 3 factors were retained and extracted for principal axis factoring. After extraction, the first three factors accounted for 67.27% of the total variance (48.33%, 13.34%, 5.60%). An oblique rotation yielded a clear simple structure: Factor 1 (perceived norms) loadings *λ* = 0.623 to .870. Factor 2 (feeling ignored) loaded *λ* = 0.472 to 0.758; and Factor 3 (interpersonal conflict) loaded *λ* = 0.682 to 0.851. Interfactor correlations were modest to moderate (*r*_12_ = 0.322, *r*_13_ = 0.631, *r*_23_ = 0.274). Rotated loadings are presented in Table 6.

**TABLE 6:** Exploratory factor analysis: Rotated factor loadings for the generic scale of being phubbed (GSBP).

Items	Perceived norms	Feeling ignored	Interpersonal conflict
GSBP5	0.713		
GSBP10	0.810		
GSBP11	0.863		
GSBP12	0.870		
GSBP13	0.849		
GSBP14	0.859		
GSBP15	0.686		
GSBP16	0.733		
GSBP17	0.623		
GSBP1		0.737	
GSBP2		0.733	
GSBP3		0.708	
GSBP4		0.758	
GSBP6		0.652	
GSBP7		0.630	
GSBP8		0.472	
GSBP9		0.522	
GSBP18			0.682
GSBP19			0.851
GSBP20			0.826
GSBP21			0.695
GSBP22			0.758

The three‐factor model for the 22 GSBP items showed marginal‐to‐acceptable overall fit: *χ* ^2^ (206) = 3100.30, *p* < 0.001; CFI = 0.928, TLI = 0.897, RMSEA = 0.083, SRMR = 0.076. All standardized loadings were significant (*p* < 0.001): Factor 1 = 0.837–0.940. (GSBP5, GSBP10‐17); Factor 2 = 0.430–0.925 (GSBP1‐4, GSBP6‐9); Factor 3 = 0.822–0.885 (GSBP18‐22). Residual variances spanned .117–0.815. The internal consistency of the scale was good (total *ω* = 0.946). McDonald’s *ω* subscale reliability was also strong with values of 0.947 for “perceived norms”, 0.893 for “feeling ignored”, and 0.898 for “interpersonal conflict”. Regarding convergent validity, total smartphone addiction measured with SAS‐SV, correlated positively with overall being phubbed (GSBP), *ρ* = 0.292, 95% CI [0.197,0.381], *p* < 0.001. Total SAS‐SV also showed moderate positive associations with the three GSBP factors: perceived norms *ρ* = 0.192, 95% CI [0.093,0.286], feeling ignored *ρ* = 0.341, 95% CI [0.250,0.427], and interpersonal conflict *ρ* = 0.188, 95% CI [0.089,0.283], all *p* < 0.001 (two‐tailed). Using the standardized loadings from the CFA, the three GSBP factors showed strong convergent evidence: AVE = 0.574–0.794 and CR = 0.911–0.972. Discriminant validity was supported by the Fornell–Larcker criterion: The diagonals √AVE = 0.758–0.891, showing that each factor shares more variance with its own indicators than with other constructs (Table 7).

**TABLE 7:** GSBP descriptive statistics, average variance extracted, and reliability analysis.

Instrument/subscale	*N*	M ± SD	AVE	√AVE	CR	*ω*
GSBP	406	3.20 ± 0.90	—	—	—	0.946
Perceived norms	406	3.97 ± 0.98	0.794	0.891	0.972	0.947
Feeling ignored	406	2.79 ± 1.11	0.575	0.758	0.911	0.893
Interpersonal conflict	406	2.48 ± 1.19	0.749	0.865	0.937	0.898

*Note: N*, frequencies; M, mean; *ω*, McDonald’s *ω*.

Abbreviations: AVE, average variance extracted; √AVE, average variance square roots; SD, standard deviation.

## 4. Discussion

The present study aimed to translate, culturally adapt, and psychometrically validate the Greek versions of the GSP and GSBP in a sample of nursing personnel in Greece. While phubbing has been examined in nursing contexts, comprehensive psychometric evidence for the GSP/GSBP specifically in nursing personnel has been limited; therefore, the present study findings extend the measurement base for research in healthcare settings. The CFA findings are broadly consistent with previous validation work showing that the GSP generally retains a stable multidimensional structure across cultural contexts [26, 36, 37]. In the present study, the GSP demonstrated adequate fit, supporting the four‐factor model in Greek nursing personnel. The GSBP showed marginal‐to‐acceptable fit, suggesting that the experience of being phubbed may be more sensitive to contextual and normative features of interpersonal interaction. This pattern is consistent with prior cross‐cultural work indicating that being phubbed may be shaped not only by the observed smartphone behavior itself but also by the social meaning attributed to that behavior. Beyond overall model fit, CFA‐derived indices provided direct evidence that the GSP and GSBP latent factors were well captured by their indicators and sufficiently distinct to justify separate subscale interpretation.

The GSP’s four‐dimensional structure was replicated in this nursing sample, supporting the conceptualization of phubbing as a multidimensional pattern rather than a single homogenous behavior. Multiple studies using CFA and second‐order factor models have validated phubbing as comprising distinct but related dimensions. Treating it as multidimensional allows for more accurate measurement, better theoretical models, and more targeted interventions [1, 37], Yam and Kumcağız [38]. Compared with the GSP, the GSBP showed a more challenging measurement structure, suggesting that perceptions of being phubbed may be more context‐ and norm‐dependent than phubbing itself. While phubbing behavior is relatively common and often habitual, the interpretation of that behavior varies widely depending on cultural background, situational norms, and interpersonal dynamics [39–41].

Internal consistency reliability in the present sample was strong for both instruments, indicating that the GSP and GSBP yield sufficiently precise scores for use in nursing research. Specifically, McDonald’s omega was good for the GSP and excellent for the GSBP, supporting stable score interpretation at both the total and subscale levels. Reliability coefficients in this range are typically considered adequate for group‐level comparisons and correlational modelling in health research [42]. These findings are consistent with the original scale development [26] which reported good internal consistency and strong short‐term stability for both scales. As an additional cross‐cultural echo, a validation study in Iran reported that both GSP and GSBP retained high internal consistency [43].

Convergent validity was supported by a coherent pattern of positive associations between SAS‐SV and both GSP and GSBP. SAS‐SV correlated moderately with overall phubbing and showed moderate associations with the GSP factors. Problematic smartphone use has been repeatedly linked to phubbing behaviors, suggesting that compulsive or dysregulated phone use can spill over into face‐to‐face interactions by increasing the tendency to attend to the device at socially inappropriate moments [44]. More broadly, a systematic meta‐analytic review of phubbing predictors concluded that problematic use patterns were the strongest predictors of phubbing, reinforcing the view that phubbing often reflects a behavioral manifestation of problematic technology engagement [45]. The stronger association between SAS‐SV and GSP compared with GSBP is theoretically meaningful. Phubbing reflects one’s own smartphone‐related behavior and is therefore expected to be more directly related to problematic smartphone use. In contrast, being phubbed reflects the perceived behavior of others and may depend more strongly on social context, relationship expectations, and workplace norms. People are more likely to tolerate or even mimic phubbing if they believe it is widespread, especially in group settings Leuppert and Geber [40].

In terms of practical implications for nursing, the culturally adapted Greek versions of the GSP and GSBP may support future research on technology‐related disruption in clinical communication, teamwork, and professional interactions. In nursing practice, digital technologies can support access to information, communication, and clinical decision‐making, but their benefits depend on appropriate and context‐sensitive use [16, 46]. When personal smartphone use interrupts face‐to‐face clinical communication, it may undermine attentiveness, responsiveness, and the quality of professional interaction [14]. From an organizational perspective, validated measures of phubbing and being phubbed may help identify technology‐related behaviors that affect workplace climate, collegial respect, and team functioning. In clinical environments where communication failures may have implications for care coordination and patient safety, understanding smartphone‐related interactional disruptions can inform educational initiatives, digital etiquette guidance, and workplace policies that support professional communication [47, 48]. Expectations regarding smartphone use during face‐to‐face communication may vary across cultural and workplace contexts; therefore, validated instruments are needed to ensure that phubbing and being phubbed are assessed in a conceptually equivalent and culturally meaningful way.

In terms of limitations, the cross‐sectional design and self‐report measurements increase the risk that observed associations are inflated by common‐method variance. The convenience sampling may limit the generalizability to other nursing contexts, i.e., different health systems, units, or organizational cultures. Also, the study did not include test‐retest reliability, so temporal stability in nurses remains to be established in Greece. Measurement invariance was not tested in the present study. Future studies should examine whether the Greek GSP and GSBP operate equivalently across key groups, including gender, nurses versus nurse assistants, hospital department, and different clinical settings. Such analyses would strengthen evidence for valid comparisons between subgroups of nursing personnel. No post hoc residual covariances were added to improve model fit, in order to preserve an a priori, theory‐consistent measurement model and avoid overfitting. In terms of strengths, the present study is the first that provides a rigorous psychometric evaluation of the Greek versions of the GSP and GSBP in the clinical nursing workforce, a population that has been underrepresented in prior validation work, using a comparatively large sample drawn from hospitals in Northern Greece. Future studies should examine the temporal stability of the Greek GSP and GSBP by employing test–retest designs. In addition, longitudinal studies are needed to establish predictive validity by testing whether GSP and GSBP scores prospectively predict outcomes such as teamwork and communication quality, interpersonal conflict, and patient‐safety‐related indicators in clinical settings.

## 5. Conclusion

Overall, the findings support the Greek versions of the GSP and the GSBP as reliable and valid measures for assessing phubbing and being phubbed among nursing personnel. The GSP demonstrated a clearer and more robust internal structure, whereas the GSBP showed acceptable psychometric performance but may be more sensitive to contextual and normative features of interpersonal interactions. Together, these tools provide a sound measurement foundation for theory‐driven research on technology‐related disruption in clinical practice communication and teamwork and for evaluating interventions aimed at promoting healthier digital etiquette in healthcare settings.

## Author Contributions

G.M.: writing–review and editing, writing–original draft, validation, supervision, resources, methodology, formal analysis, data curation, and conceptualization. E.V.: writing–review and editing, writing–original draft, supervision, resources, and data curation. V.G.: writing–review and editing, writing–original draft, resources, formal analysis, data curation, and conceptualization.

## Funding

This research did not receive any specific grant from funding agencies in the public, commercial, or nonprofit sectors.

The publication of this article in OA mode was financially supported by HEAL‐Link.

## Ethics Statement

The study was conducted in accordance with the ethical standards of the institutional research committee and with the 1964 Helsinki Declaration and its later amendments. Approval was obtained from the Ethics Committee (55,705/475–15/4/2025) prior to data collection. Participation was voluntary, and all participants provided informed consent after receiving detailed information about the study’s purpose and procedures. Confidentiality and anonymity of the respondents were strictly maintained throughout the research process**.**

## Conflicts of Interest

The authors declare no conflicts of interest.

## Data Availability

The data that support the findings of this study are available from the corresponding author upon reasonable request.

## References

[1] Chotpitayasunondh V. and Douglas K. M. , How “Phubbing” Becomes the Norm: The Antecedents and Consequences of Snubbing Via Smartphone, Computers in Human Behavior. (2016) 63, 9–18, 10.1016/j.chb.2016.05.018.

[2] Khoo S. S. and Yang H. , Smartphone Addiction and Checking Behaviors Predict Aggression: A Structural Equation Modeling Approach, International Journal of Environmental Research and Public Health. (2021) 18, no. 24, 10.3390/ijerph182413020. PMC8700868 34948631

[3] Aktipis A. , Whitaker R. , and Ayers J. D. , Do Smartphones Create a Coordination Problem for Face-to-Face Interaction? Leveraging Game Theory to Understand and Solve the Smartphone Dilemma, BioEssays. (2020) 42, no. 4, 10.1002/bies.201800261. 32130740

[4] Hayran C. and Anik L. , Well-Being and Fear of Missing Out (FOMO) on Digital Content in the Time of COVID-19: A Correlational Analysis Among University Students, International Journal of Environmental Research and Public Health. (2021) 18, no. 4, 10.3390/ijerph18041974. PMC7922067 33670639

[5] Labrague L. J. and de Los Santos J. A. A. , COVID-19 Anxiety, Coping Strategies, and Burnout Among Frontline Nurses: A Cross-Sectional Study, Journal of Nursing Management. (2021) 29, no. 5, 1262–1271, 10.1111/jonm.13282.

[6] Lai J. , Ma S. , Wang Y. et al., Factors Associated With Mental Health Outcomes Among Health Care Workers Exposed to Coronavirus Disease 2019, Jama Network Open. (2020) 3, no. 3, 10.1001/jamanetworkopen.2020.3976. PMC7090843 32202646

[7] Blanuša J. , Barzut V. , and Knežević J. , Intolerance of Uncertainty and Fear of COVID-19 Moderating Role in Relationship Between Job Insecurity and Work-Related Distress in the Republic of Serbia, Frontiers in Psychology. (2021) 12, 10.3389/fpsyg.2021.647972. PMC8226083 34177703

[8] Dozois D. J. A. , Anxiety and Depression in Canada During the COVID-19 Pandemic: A National Survey, Canadian Psychology. (2021) 62, no. 1, 136–142, 10.1037/cap0000251.

[9] Aqtam I. , Ayed A. , Toqan D. et al., The Relationship Between Stress and Resilience of Nurses in Intensive Care Units During the COVID-19 Pandemic, Inquiry: The Journal of Health Care Organization, Provision, and Financing. (2023) 60, 10.1177/00469580231179876. PMC10247682 37278278

[10] Glassman J. , Humphreys K. , Yeung S. et al., Parents’ Perspectives on Using Artificial Intelligence to Reduce Technology Interference During Early Childhood: Cross-Sectional Online Survey, Journal of Medical Internet Research. (2021) 23, no. 3, 10.2196/19461. PMC8074848 33720026

[11] Karadağ E. , Tosuntaş Ş. B. , Erzen E. et al., Determinants of Phubbing, Which is the Sum of Many Virtual Addictions: A Structural Equation Model, Journal of Behavioral Addictions. (2015) 4, no. 2, 60–74, 10.1556/2006.4.2015.005. 26014669 PMC4500886

[12] Şafak A. and Oflaz F. , Evaluation of Phubbing, Online Social Support and Trait Anxiety in Nurses, International Nursing Review. (2024) 71, no. 4, 794–800, 10.1111/inr.12916. 38054568 PMC11600543

[13] Gao B. , Shen Q. , Luo G. , and Xu Y. , Why Mobile Social Media-Related Fear of Missing out Promotes Depressive Symptoms? the Roles of Phubbing and Social Exclusion, BMC Psychology. (2023) 11, no. 1, 10.1186/s40359-023-01231-1. PMC10311784 37386513

[14] Fiorinelli M. , Di Mario S. , Surace A. et al., Smartphone Distraction During Nursing Care: Systematic Literature Review, Applied Nursing Research: ANR. (2021) 58, 10.1016/j.apnr.2021.151405. 33745553

[15] Yousaf S. , Balkaş J. , Taş M. et al., Boss Phubbing and Its Consequences in the Hospitality Industry: A Moderated Mediation Model, Journal of Business Research. (2022) 142, 194–204, 10.1016/j.jbusres.2022.01.043.

[16] De Jong A. , Donelle L. , and Kerr M. , Nurses’ Use of Personal Smartphone Technology in the Workplace: Scoping Review, JMIR mHealth and uHealth. (2020) 8, no. 11, 10.2196/18774. PMC7728531 33242012

[17] Muñoz-Carril P.-C. , Bargiela I. M. , Estévez I. , and Bonilla-Del-Río M. , Analysis of Phubbing Among University Students: A Study of Its Prevalence, Incidence Factors and Predictors, European Journal of Investigation in Health Psychology and Education. (2025) 15, no. 10, 10.3390/ejihpe15100201. PMC12563129 41149152

[18] Cho S. and Lee E. , Distraction by Smartphone Use During Clinical Practice and Opinions About Smartphone Restriction Policies: A Cross-Sectional Descriptive Study of Nursing Students, Nurse Education Today. (2016) 40, 128–133, 10.1016/j.nedt.2016.02.021. 27125162

[19] McBride D. L. , LeVasseur S. A. , and Li D. , Non-Work-Related Use of Personal Mobile Phones by Hospital Registered Nurses, JMIR mHealth and uHealth. (2015) 3, no. 1, 10.2196/mhealth.4001. PMC4319148 25586982

[20] McBride D. L. , LeVasseur S. A. , and Li D. , Nursing Performance and Mobile Phone Use: Are Nurses Aware of Their Performance Decrements?, JMIR Human Factors. (2015) 2, no. 1, 10.2196/humanfactors.4070. PMC4797662 27026182

[21] David M. E. and Roberts J. A. , Developing and Testing a Scale Designed to Measure Perceived Phubbing, International Journal of Environmental Research and Public Health. (2020) 17, no. 21, 10.3390/ijerph17218152. PMC7663168 33158203

[22] Koçak Ö. E. , Being Phubbed in the Workplace: A New Scale and Implications for Daily Work Engagement, Current Psychology. (2021) 40, no. 12, 6212–6226, 10.1007/s12144-021-01635-5.

[23] Pancani L. , Gerosa T. , Gui M. , and Riva P. , Mom, Dad, Look at me: The Development of the Parental Phubbing Scale, Journal of Social and Personal Relationships. (2021) 38, no. 2, 435–454, 10.1177/0265407520964866.

[24] Roberts J. A. and David M. E. , My Life Has Become a Major Distraction From my Cell Phone: Partner Phubbing and Relationship Satisfaction Among Romantic Partners, Computers in Human Behavior. (2016) 54, 134–141, 10.1016/j.chb.2015.07.058.

[25] Roberts J. A. and David M. E. , Put Down Your Phone and Listen to me: How Boss Phubbing Undermines the Psychological Conditions Necessary for Employee Engagement, Computers in Human Behavior. (2017) 75, 206–217, 10.1016/j.chb.2017.05.021.

[26] Chotpitayasunondh V. and Douglas K. M. , Measuring Phone Snubbing Behavior: Development and Validation of the Generic Scale of Phubbing (GSP) and the Generic Scale of Being Phubbed (GSBP), Computers in Human Behavior. (2018) 88, 5–17, 10.1016/j.chb.2018.06.020.

[27] Kaynar Demirel N. S. and Basan İ. N. , The Relationship Between Nursing Students’ Attitudes Toward E-Learning and Phubbing Behavior: A Descriptive and Correlational Study, Journal of Education and Research in Nursing. (2025) 22, no. 4, 239–244, 10.14744/jern.2025.47124.

[28] Karaduman G. S. , Basak T. , Santana Fialho Sim-Sim M. M. , Aaberg V. , and Bule M. J. , Nomophobia and Phubbing Levels of Nursing Students: A Multicenter Study, Computers, Informatics, Nursing. (2024) 42, no. 8, 601–607, 10.1097/CIN.0000000000001154. 38832877

[29] Başgöl Ş. and Rüzgar Ş. , The Effect of Midwifery Students’ Phubbing Behaviors on Communication Skills in Türkiye, Nurse Education in Practice. (2025) 85, 10.1016/j.nepr.2025.104382. 40319795

[30] Kaviani F. , Robards B. , Young K. L. , and Koppel S. , Nomophobia: Is the Fear of being Without a Smartphone Associated With Problematic Use?, International Journal of Environmental Research and Public Health. (2020) 17, no. 17, 10.3390/ijerph17176024. PMC7504166 32824979

[31] Kamtsios S. , Kakouris V. , and Volioti S. , Personality Traits and Undergraduates’ Phubbing Behavior, Hellenic Journal of Psychology. (2025) 22, no. 2, 160–191, 10.26262/hjp.v22i2.10442.

[32] MacCallum R. C. , Widaman K. F. , Zhang S. , and Hong S. , Sample Size in Factor Analysis, Psychological Methods. (1999) 4, no. 1, 84–99, 10.1037/1082-989X.4.1.84.

[33] Kwon M. , Kim D.-J. , Cho H. , and Yang S. , The Smartphone Addiction Scale: Development and Validation of a Short Version for Adolescents, PLoS One. (2013) 8, no. 12, 10.1371/journal.pone.0083558. PMC3877074 24391787

[34] Manomenidis G. , Karavasileiadou S. , Pafis K. , and Vasileiou E. , Psychometric Properties of the Smartphone Addiction Scale—Short Version Among Nursing Students in Greece, Psychiatry International. (2026) 7, no. 3, 10.3390/psychiatryint7030098.

[35] Beaton D. E. , Bombardier C. , Guillemin F. , and Ferraz M. B. , Guidelines for the Process of Cross-Cultural Adaptation of Self-Report Measures, Spine. (2000) 25, no. 24, 3186–3191, 10.1097/00007632-200012150-00014. 11124735

[36] Bitar Z. , Akel M. , Salameh P. , Obeid S. , and Hallit S. , Phubbing Among Lebanese Young Adults: Scale Validation and Association With Mental Health (Depression, Anxiety, and Stress), Current Psychology. (2022) Advance online publication, New Brunswick, N.J, 1–12, 10.1007/s12144-022-03104-z. PMC9039595 35496363

[37] Mento C. , Silvestri M. C. , Lombardo C. et al., Phubbing and Phubber Behavior: A New Perspective in Clinical Psychological Assessment, AIMS Public Health. (2025) 12, no. 3, 716–734, 10.3934/publichealth.2025037. 41127430 PMC12538237

[38] Yam F. C. and Kumcağız H. , Adaptation of General Phubbing Scale to Turkish Culture and Investigation of Phubbing Levels of University Students in Terms of Various Variables, Addicta: The Turkish Journal on Addictions. (2020) 7, no. 1, 48–60.

[39] Büttner C. M. , Albath E. A. , and Greifeneder R. , Cultural Differences in Perceiving Co-Present Phone Use as Phubbing: Evidence From Six Countries, Social Influence. (2025) 20, no. 1, 10.1080/15534510.2024.2447275.

[40] Leuppert R. and Geber S. , Commonly Done but Not Socially Accepted? Phubbing and Social Norms in Dyadic and Small Group Settings, Communication Research Reports. (2020) 37, no. 3, 55–64, 10.1080/08824096.2020.1756767.

[41] Mosley M. A. and Parker M. L. , The Phubbing Problem: A Dyadic Exploration of the Moderating Role of Partner Phubbing on Attachment and Couple Satisfaction, Computers in Human Behavior. (2023) 149, 1–13, 10.1016/j.chb.2023.107953.

[42] Boateng G. O. , Neilands T. B. , Frongillo E. A. , Melgar-Quiñonez H. R. , and Young S. L. , Best Practices for Developing and Validating Scales for Health, Social, and Behavioral Research: A Primer, Frontiers in Public Health. (2018) 6, 10.3389/fpubh.2018.00149. PMC6004510 29942800

[43] Akbari M. , Seydavi M. , Rahmati S. , and Wright P. J. , Psychometric Validation of the Persian Versions of the Generic Scale of Phubbing (GSP) and the Generic Scale of Being Phubbed (GSBP): Factor Structure, Reliability, and Construct Validity in an Iranian Community Sample, Current Psychology. (2026) 45, no. 1, 10.1007/s12144-025-08785-w.

[44] Nie J. , Wang P. , and Lei L. , Why Can’t we be Separated From Our Smartphones? the Vital Roles of Smartphone Activity in Smartphone Separation Anxiety, Computers in Human Behavior. (2020) 109, 10.1016/j.chb.2020.106351.

[45] Arenz A. and Schnauber-Stockmann A. , Who “Phubs”? A Systematic Meta-Analytic Review of Phubbing Predictors, Mobile Media & Communication. (2023) 12, no. 3, 637–661, 10.1177/20501579231215678.

[46] Batran A. , Al-Humran S. M. , Malak M. Z. , and Ayed A. , The Relationship Between Nursing Informatics Competency and Clinical Decision-Making Among Nurses in West Bank, Palestine, Computers, Informatics, Nursing. (2022) 40, no. 8, 547–553, 10.1097/CIN.0000000000000890. 35234705

[47] Ayed A. , Abu Ejheisheh M. , Aqtam I. , Batran A. , and Farajallah M. , The Relationship Between Professional Quality of Life and Work Environment Among Nurses in Intensive Care Units, Inquiry: The Journal of Health Care Organization, Provision, and Financing. (2024) 61, 10.1177/00469580241297974. PMC11550503 39520216

[48] Batran A. , Aqtam I. , Ayed A. , and Abu Ejheisheh M. , The Relationship Between Professional Quality of Life and Work Environment Among Nurses in Neonate Care Units, PLoS One. (2025) 20, no. 4, 10.1371/journal.pone.0322023. PMC12027106 40279366

[49] Butt A. K. and Arshad T. , The Relationship Between Basic Psychological Needs and Phubbing: Fear of Missing out as the Mediator, Psychiatry and Clinical Psychopharmacology. (2021) 10, no. 6, 916–925, 10.1002/pchj.483. 34510810

